# Strong coupling in metal-semiconductor microcavities featuring Ge quantum wells: a perspective study

**DOI:** 10.1515/nanoph-2023-0730

**Published:** 2024-01-24

**Authors:** Marco Faverzani, Stefano Calcaterra, Paolo Biagioni, Jacopo Frigerio

**Affiliations:** Politecnico di Milano, Milano, Italy

**Keywords:** strong coupling, quantum wells, germanium

## Abstract

In this work we theoretically investigate the possibility of observing strong coupling at mid-infrared frequencies within the group-IV semiconductor material platform. Our results show that the strong coupling condition is attainable in Ge/SiGe quantum wells integrated in hybrid metal-semiconductor microcavities, featuring a highly n-doped SiGe layer as one of the mirrors.

## 1 Introduction

Intersubband (ISB) transitions in semiconductor quantum wells (QWs) have drawn a lot of attention because of their potential application in optoelectronic devices working in the mid- and far-infrared spectral regions down to the THz. In the last 30 years, this paved the way for the development of quantum cascade lasers (QCLs) [1] and of infrared detectors either operating in photoconductive mode such as quantum well infrared photodetectors (QWIPs) [2] or in photovoltaic mode such as quantum cascade detectors (QCDs) [3].

Indeed, quasi-particles known as ISB polaritons emerge when a strong interaction between ISB transitions and photonic modes in microcavities is established [4]–[7]. Such ISB polaritons are not only interesting for fundamental physics but they also allow for the implementation of devices with improved performances. ISB polaritonic devices include ISB polariton emitters [8], [9], QWIPs and QCDs operating in the strong coupling regime [10], [11], non-linear devices [12], [13], and modulators [14].

The physics of ISB polaritons has been deeply studied since their first observation [4]. However, most of these studies focused on multiple quantum well (MQW) structures made of III-V semiconductor alloys, leaving group-IV-based systems almost unexplored. Silicon, germanium and their alloys have recently gained interest because of the opportunity of monolithically integrating devices with classical electronic circuits [15]. The optical properties of Si/SiGe and Ge/SiGe QWs have been widely investigated in the past, both theoretically [16], [17] and experimentally [18]–[20] for their high technological potential. The epitaxial growth of SiGe heterostructures has undergone a remarkable technological development over the last decade, mainly driven by their prospective applications in silicon photonics [21]–[25]. The possibility of realizing nanometre-thick Ge wells with very high crystalline quality [26], [27] has paved the way toward their exploitation for mid- [28], [29] and far-infrared photonics [30], [31]. In this work, we study the interaction between ISB transitions in silicon–germanium MQW structures and the photonic modes of square patch antenna arrays resonant in the mid-infrared (MIR) to assess the possibility of reaching the strong coupling condition.

We consider hole-doped MQWs because of the larger band offset achievable in the valence band with respect to the conduction band, resulting in more spaced energy levels and thus ISB transitions occurring in the MIR. In addition, we assess the possibility of using a heavily-doped SiGe epilayer as the bottom mirror of the cavity instead of a metal. Although being characterized by larger losses, this approach has the potential to strongly simplify the technological implementation of ISB polaritonic devices, since it eliminates the need for more complex fabrication processes such as substrate removal.

The paper is organized as follows: in Section 2 we will introduce the sample under investigation, in Section 3 we will describe the simulations that we carried out and we will analyse the obtained results and, finally, in Section 4 we will draw the conclusions and discuss some possible future developments.

## 2 Structure and optical properties of the sample

In order to assess the possibility of entering the strong-coupling regime with hole-doped Ge/SiGe QWs, we consider a square QW designed to provide an ISB transition in the MIR at *λ* ≃ 8.5 µm for TM-polarized light. The choice of a simple, non-optimized QW design aims at maintaining the highest level of generality.

**Figure 1:**
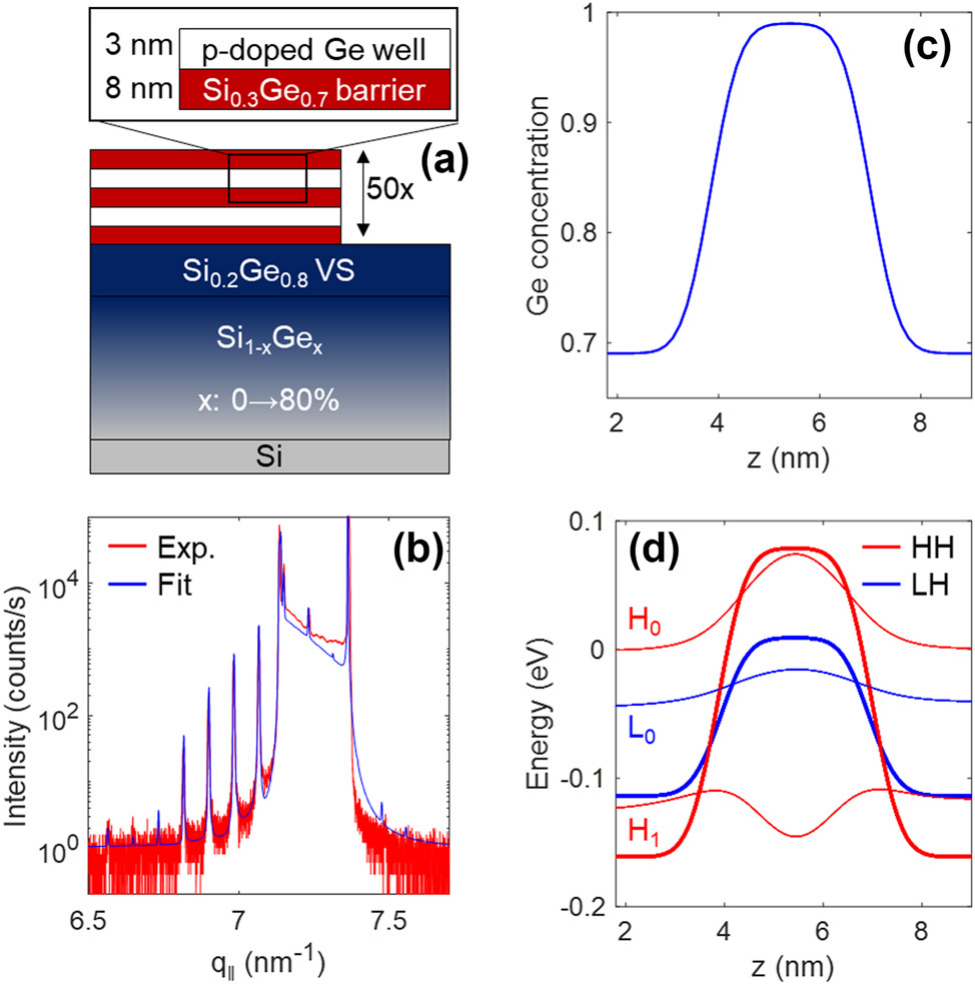
Structure and energy levels of the investigated heterostructure. (a) Pictorial representation of the nominal structure of the sample. (b) Experimental and simulated X-ray diffraction scans. (c) Ge-content profile as retrieved by X-ray diffraction measurements. (d) Potential profile and wavefunctions of the confined energy levels calculated with the nextnano software.

The sample has been grown by means of low-energy-plasma-enhanced chemical vapor deposition (LEPECVD) [32] on a 100 mm p-Si $\left(001\right)$ substrate. The first part of the structure consists of a graded buffer, where the Ge concentration was raised linearly from 0 % to 80 % with a grading rate of 7 %/µm. Then a fully relaxed 2 µm-thick layer of Si_0.2_Ge_0.8_ was grown to serve as a virtual substrate (VS) for the growth of the quantum wells. The MQW stack, which has been grown at 400 °C at a rate of 10 nm/min, consists of 50 repetitions of an 8 nm-thick Si_0.3_Ge_0.7_ barrier and a 3 nm-thick Ge quantum well. The Ge wells have been p-doped with boron by adding B_2_H_6_ during the growth, targeting a doping density of 7 × 10^11^ cm^−2^. A sketch of the sample showing the relevant epitaxial steps is shown in Figure 1(a).

The sample has been characterized by high-resolution X-ray diffraction (HR-XRD) measurements with a PANalytical X’Pert PRO MRD diffractometer. Figure 1(b) shows the $\left(004\right)$ *ω*–2*θ* scan, superimposed to multi-beam dynamical Darwin model simulations [33], [34] as implemented in the xrayutilities package [35]. The QW compositional profile retrieved by fitting the experimental XRD data is shown in Figure 1(c). A Gaussian smoothening of the QW ideal profile has been introduced to model the interdiffusion occurring at the Ge/SiGe interfaces. This assumption allowed to fit both the low and the high-order diffraction peaks. The band structure of the sample has been calculated by means of 8-band k·p modelling as implemented in the nextnano software package, using the experimentally retrieved Ge profile as an input parameter. The potential energy profiles at the valence band maximum for the HH and LH bands are reported in Figure 1(d), together with the related wavefunctions. The plot has been rescaled to have the ground state at 0 eV. The heterostructure features three confined states: the ground state H_0_, the L_0_ state around 45 meV and the H_1_ state around 140 meV.

**Figure 2:**
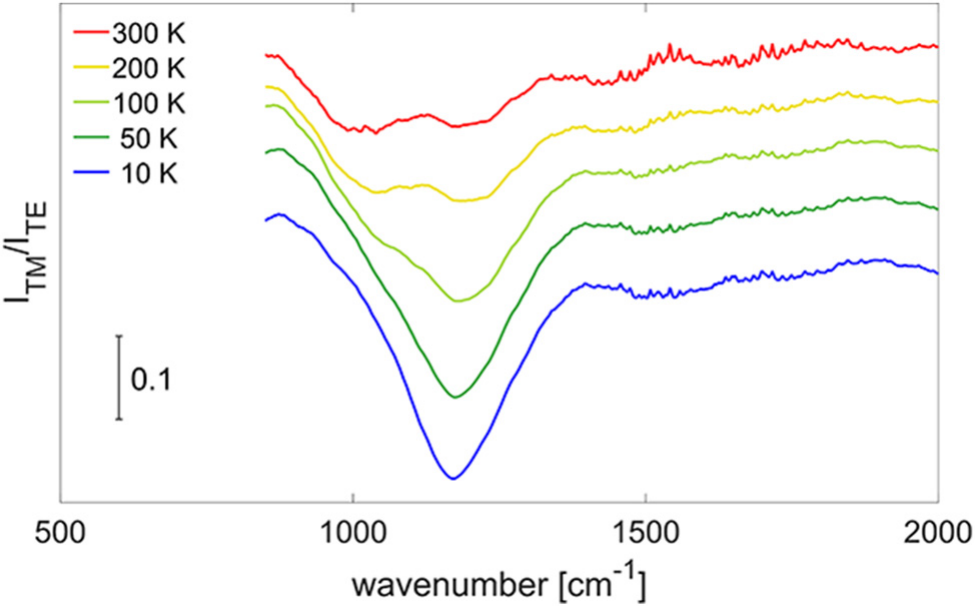
Temperature-dependent dichroic transmission spectra of the ISB transition in the MQW under study. The temperature was swept between 10 and 300 K. The spectra are displaced along the vertical direction for clarity.

To determine the energy and the line-shape of the ISB absorption, dichroic transmission spectra were collected with a commercial Fourier-transform infrared (FTIR) spectrometer. The sample was cut in the common prism-like multi-pass waveguide geometry and two transmission spectra were acquired for TM- and TE-polarized light, respectively. The dichroic ratio *I*_TM_/*I*_TE_ between the two spectra was then computed to get rid of the contributions coming from the substrate and emphasize the feature related to the TM-polarized ISB absorption. Figure 2 shows the measured spectra as a function of the temperature. To retrieve the transition energy and the spectral width of the ISB absorption, a Lorentzian fit was employed. At low temperature, the fitting yields a transition energy around 145 meV (1170 cm^−1^) and a full width at half maximum around 24 meV (200 cm^−1^).

To determine the two-dimensional hole density inside the wells, we first compute the two-dimensional absorption coefficient from the dichroic transmission by applying the relation [2]

$$\alpha_{2\text{D}}=\frac{-\ln\left(I_{\text{TM}}/I_{\text{TE}}\right)}{CMN_{\text{qw}}}\frac{\cos\theta }{\sin^{2}\theta }$$

where *θ* is the angle at which the light impinges onto the quantum wells, *N*_qw_ is the number of periods contained in the MQW stack, *M* is twice the number of reflections occurring at the gold layer and *C* ≃ 1.5 is a parameter which takes into account the enhancement of the *z* component of the electric field due to the presence of gold. The two-dimensional charge density *n*_2D_ can then be retrieved using the equation [2]

$$\int \alpha_{2\text{D}}\left(E\right)\text{d}E=n_{2D}\frac{\pi e^{2}\hbar f_{1\to 2}}{2m^{*}c\varepsilon_{0}n_{\text{eff}}}$$

where *f*_1→2_, m* and *n*_eff_ are the oscillator strength of the 1 → 2 ISB transition, the effective mass of the charge carriers and the effective index of the MQW. At first order, we can compute the oscillator strength as if the well were infinite: the 1 → 2 transition is in this case characterized by an oscillator strength *f*_1→2_ ≃ 0.96 [2] which is almost unity and will be therefore neglected in the following. Moreover, the effective mass of the carriers is approximately equal to that of heavy holes in bulk germanium, i.e. m* ≃ 0.33 *m*_*e*_. Indeed, the ground state belongs to the heavy-hole band because of the compressive strain in the Ge well. Finally, the effective index of the MQWs can be computed with an effective-medium approach as the integral average of the spatial-dependent dielectric function describing the MQW stack. In doing so, we estimate the high-frequency dielectric constant of the Si_1-*x*_Ge_*x*_ alloy as the weighted average of the infrared permittivity of silicon, i.e. *ɛ*_Si_ = 11.7, and germanium, i.e. *ɛ*_Ge_ = 16.2, obtaining

(1) $$\varepsilon_{\text{S}{\text{i}}_{1-x}\text{G}{\text{e}}_{x}}=11.7\left(1-x\right)+16.2x.$$

This procedure returns an effective index *n*_eff_ ≃ 3.9 and a two-dimensional charge density around 7 × 10^11^ cm^−2^ for the MQWs under investigation.

To the aim of realizing the patch antenna microcavities which should couple to the ISB transition, we need to enclose the MQW stack within two highly-reflective layers: while the top layer for the fabrication of the patch antennas can be easily obtained by depositing a Ti/Au layer, a similar gold-based bottom mirror would require removing the substrate below the MQWs. For this reason, we explore here the possibility of doping the Si_0.2_Ge_0.8_ constant composition layer in such a way that the spectral region of interest lies at energies below the screened plasma frequency, where the reflectivity of the semiconducting mirror is high enough. Within this approach, this epilayer serves both as the bottom mirror as well as the VS for the MQWs.

## 3 FDTD simulation of the sample

The simulations presented in this work were performed with the FDTD solver by Ansys Lumerical and focused on the wavelength region between 5 and 20 μm. The physical quantity we are interested in is the normal-incidence reflectivity of the sample which was acquired with plane wave illumination and a frequency domain power monitor placed above the whole structure.

### 3.1 n-doped SiGe mirror

For the reasons that we have mentioned in the previous section, the lower mirror was modelled as an n-doped Si_0.2_Ge_0.8_ layer whose dielectric function follows the well-known Drude equation

$$\varepsilon_{\text{drude}}\left(\tilde{\nu }\right)=\varepsilon_{\infty }-\frac{{\tilde{\nu }}_{p}^{2}}{{\tilde{\nu }}^{2}+i\gamma \tilde{\nu }}.$$

At first order, we can neglect the losses *γ* and it can be easily seen that the material would behave as a metal when $\tilde{\nu }<{\tilde{\nu }}_{p}/\sqrt{\varepsilon_{\infty }}$ : hence, if we want the material to be a mirror at wavenumbers below 2000 cm^−1^, the plasma frequency should be as high as 7500 cm^−1^. Being Si_0.2_Ge_0.8_ Si-like, the effective mass of the conduction electrons is similar to that of silicon, i.e. $m_{e}^{*}≃0.26m_{e}$ , and this implies that a free electron density of almost 2 × 10^20^ cm^−3^ is required. Such high doping level has already been demonstrated experimentally for pure germanium [36]. In light of this discussion, we considered in the simulations an n-doped Si_0.2_Ge_0.8_ layer with a free carrier concentration equal to 2 × 10^20^ cm^−3^.

**Figure 3:**
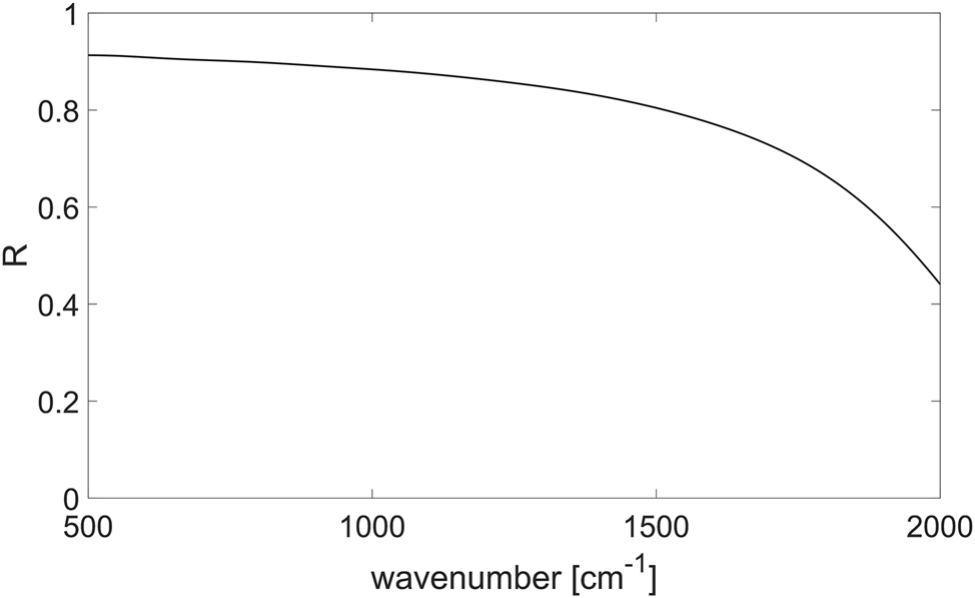
Simulated reflectivity spectrum of an n-doped Si_0.2_Ge_0.8_ film deposited on the graded buffer on silicon substrate.

The losses *γ* for such a high concentration are set to 380 cm^−1^, a value which has been inferred from experimental reflectivity measurements [36]. The corresponding scattering time *τ* = 14 fs is also in line with other works present in the literature [37]. The simulated reflectivity of the n-doped Si_0.2_Ge_0.8_ layer on top of the graded buffer and of the silicon substrate is reported in Figure 3.

### 3.2 Metal-dielectric microcavity

Before considering the coupling regime, let us consider what happens when the SiGe MQWs are not optically active, i.e. when they are not doped. Without ISB absorption, the dominant absorption mechanisms are mainly related to the lossy nature of the heavily-doped SiGe mirror.

**Figure 4:**
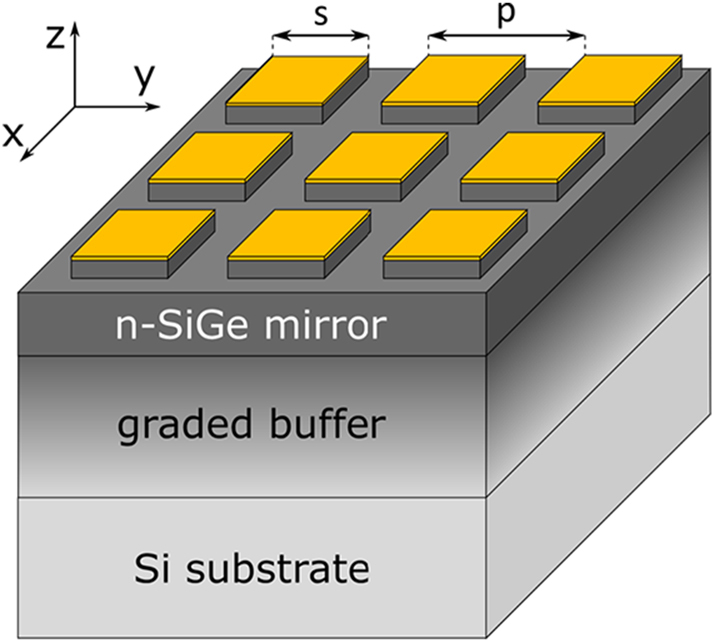
Sketch of the sample under investigation.

Neglecting the ISB contribution to the dielectric function, the infrared optical properties of the MQWs do not depend on the frequency. Therefore, the effective dielectric constant of the MQWs can be simply defined by evaluating equation (1) with the average germanium content of the heterostructure. To simulate the impact of the cavity modes on the reflectivity of the system, the MQWs were therefore described by the effective dielectric constant $\varepsilon_{\infty }^{\text{MQW}}=15.3$ . The microcavity array that we have analysed consists of square-shaped microcavities where the MQW layer has been etched except below the gold patches [38], as shown in Figure 4. The features associated to the modes of such microcavities can be tuned by playing with the geometrical parameters defining the array, e.g. the size *s* of the square gold antennas, their periodicity *p* and the total thickness of the MQW stack.

**Figure 5:**
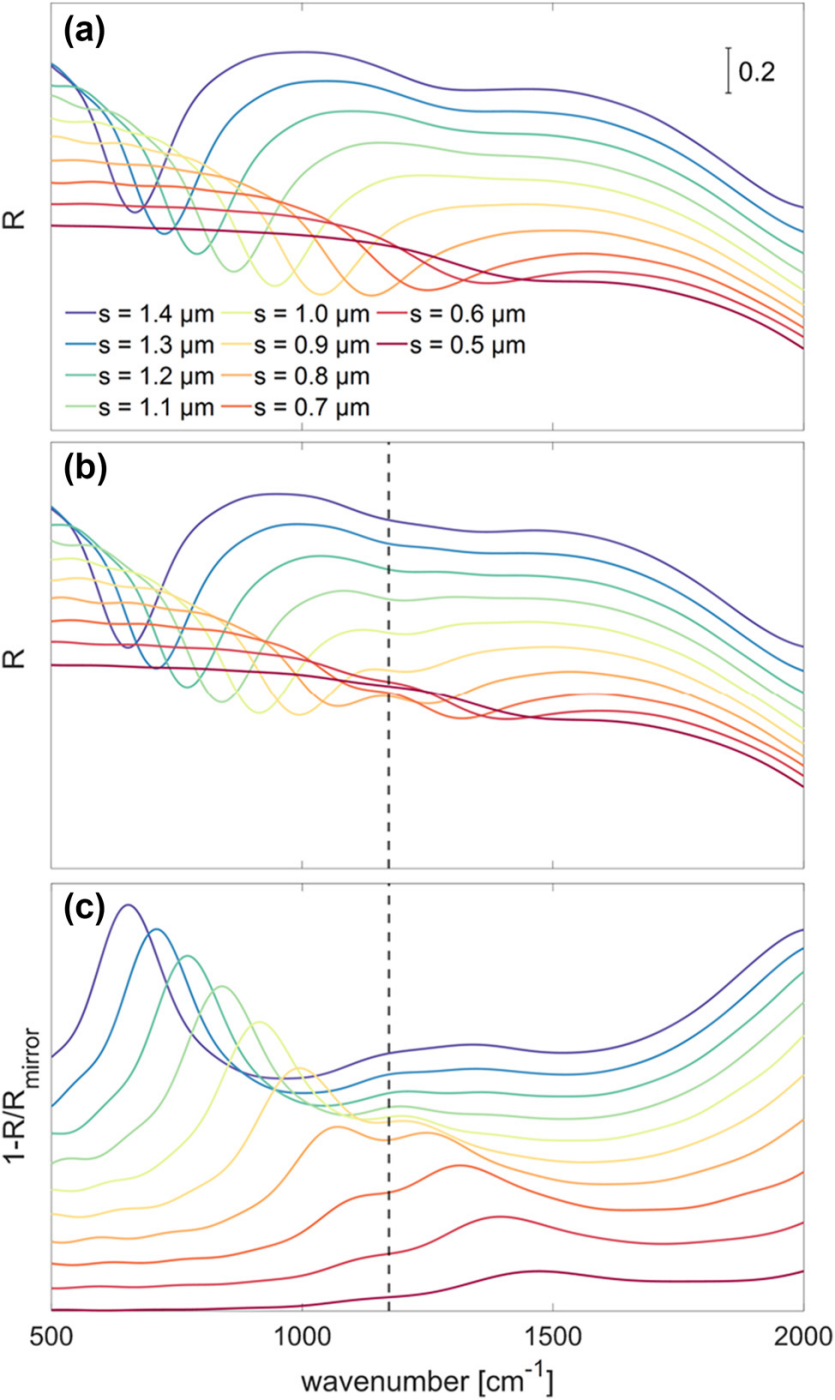
Simulated reflectivity for different antenna sizes at a fixed periodicity of 2 µm of (a) the bare square-shaped microcavity and of (b) the square-shaped microcavity coupled with the ISB transition. (c) Reflectivity spectra acquired in the coupling regime normalized to the reflectivity of the SiGe mirror subtracted from unity for convenience.

The lateral size *s* of the gold patch antenna is the parameter which mainly influences the frequency of the modes. In particular, the microcavity resonance red-shifts with increasing *s* as it can be clearly seen in Figure 5(a). The periodicity *p*, instead, determines the portion of the SiGe mirror which is covered by the antennas and thus the amount of absorbed power for a given illumination area. The choice *p* = 2 μm, which is possibly far from being optimized, is therefore made to achieve a good visibility of the spectral absorption dip while still maintaining the neighbouring antennas in a non-interacting regime. It is also worth mentioning that, besides the fundamental antenna mode, another weaker absorption dip appears around 1300 cm^−1^ in some of the spectra of Figure 5(a) for the largest patch antennas. Through a modal and field-distribution analysis of the simulation results, we attribute it to a Fabry-Pérot resonance associated with a guided mode supported by the MQW slab, with propagation direction parallel to the sample surface. Concerning the choice of the total thickness of the MQW stack, it must be borne in mind that, when this increases, further Fabry-Pérot resonances become relevant. Therefore, since this work is intended to assess the observability of the strong coupling regime in group-IV heterostructures, we decided to set the height in such a way that only one resonance can be observed in the spectral region of interest.

With the following simulations, we aim at observing how the ISB transitions and the cavity resonances are modified when their coupling is considered. For this purpose, we need to tune the resonance of the cavity to the spectral region around the ISB transition energy. Hence, we decided to consider patch antenna sizes between 0.5 and 1.4 μm and the thickness of the MQW stack was set to 250 nm. Moreover, for the reasons that we have previously discussed, we opted for a periodicity of 2 μm.

### 3.3 Strong coupling regime

At this point, to observe ISB polaritons, we still need to introduce the effect of ISB absorption into the simulations. ISB transitions are polarization-dependent and, in particular, the transition we are interested in can only be observed with light polarized along the growth direction of the MQWs. As a consequence, the MQWs cannot be described by an isotropic dielectric function but rather by a diagonal dielectric tensor. Moreover, within the effective medium approach that we have assumed before, the local variations of the permittivity can be neglected because they occur on a length scale which is much smaller than the wavelength of the photons of the impinging light. The ISB absorption, which only influences the *z* component of the dielectric tensor, is then accounted for by means of a Lorentzian function [39], [40]. Thus, the tensor that we employed in our simulations is written as

$$\varepsilon_{\text{MQW}}\left(\tilde{\nu }\right)=\varepsilon_{\infty }^{\text{MQW}}\left[\begin{array}{ccc} 1 & 0 & 0\\ 0 & 1 & 0\\ 0 & 0 & 1+\frac{{\tilde{\nu }}_{p}^{2}}{{\tilde{\nu }}_{\text{ISB}}^{2}-{\tilde{\nu }}^{2}-i\gamma_{\text{ISB}}\tilde{\nu }} \end{array}\right]$$

where $\varepsilon_{\infty }^{\text{MQW}}=15.3$ as for the optically inactive MQWs, ${\tilde{\nu }}_{\text{ISB}}$ and *γ*_ISB_ are the frequency and the half width at half maximum of the ISB transition and

$${\tilde{\nu }}_{p}=\frac{1}{2\pi c}\sqrt{\frac{f_{1\to 2}n_{2\text{D}}e^{2}}{m^{*}\varepsilon_{0}\varepsilon_{\infty }^{\text{MQW}}L_{\text{eff}}}}$$

with *L*_eff_ being the effective length scale over which the carriers spread, approximately equal to the total well width, i.e. 3 nm. As it has been already discussed, the holes mainly live in the germanium wells and their effective mass is thus approximately m* ≃ 0.33 *m*_*e*_. If we finally assume the carrier density that we have estimated in Section 2, i.e. 7 × 10^11^ cm^−2^, the plasma frequency ${\tilde{\nu }}_{p}$ turns out to be around 200 cm^−1^. The resonance energy and the broadening of the Lorentzian function are directly taken from the measurements reported in Figure 2 and they are 1170 cm^−1^ and 200 cm^−1^ respectively at cryogenic temperatures.

After having added the ISB contribution to the optical properties of the MQWs, we ran a new set of simulations and we obtained the reflectivity of the whole structure shown in the spectra of Figure 5(b). It can be noted that two dips are now visible: the two features lay in two different regions separated by the ISB transition energy and they show the anti-crossing behaviour typical of the strong coupling regime.

To better appreciate the dispersion of the frequency of the two peaks as a function of the antenna size, we tried to get rid of the non-flat background by normalizing it to the reflectivity of the SiGe mirror: Figure 5(c) illustrates the spectra obtained in this way which were then subtracted from unity for the sake of convenience.

**Figure 6:**
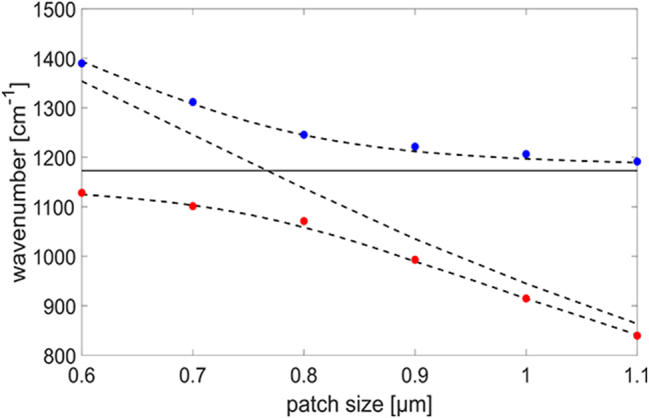
Upper (blue) and lower (red) polariton peak frequency as a function of the patch antenna size for a periodicity of 2 μm. The numerically computed frequency of the mode of the bare cavity is also reported as well as the theoretical dispersion relation of the polaritonic peaks retrieved by the fitting.

From these spectra, it is possible to identify the position of the two peaks as a function of the antenna size. Actually, if we look more carefully at the spectra corresponding to antennas of lateral size between 1.2 and 1.4 μm, it could be noticed that there is a further splitting, likely related to the coupling between the ISB transition and the resonant Fabry–Pérot absorption mechanism which is responsible for the dip around 1300 cm^−1^ that we have already mentioned when discussing the bare metal–dielectric microcavity in Figure 5(a). Moreover, in the spectrum of the *s* = 0.5 μm antenna, the lower-polariton peak can be hardly distinguished. For these reasons, we only used the data for patch antennas of size between 0.6 and 1.1 μm to draw the dispersion relation which is shown in Figure 6.

The dispersion relation of Figure 6 can be theoretically described by the secular equation [41]

$$\left({\tilde{\nu }}^{2}-{\tilde{\nu }}_{\text{ISB}}^{2}-{\tilde{\nu }}_{p}^{2}\right)\left({\tilde{\nu }}^{2}-{\tilde{\nu }}_{\text{cavity}}^{2}\right)=\Gamma f_{w}{\tilde{\nu }}_{p}^{2}{\tilde{\nu }}_{\text{cavity}}^{2}$$

where Γ is a factor accounting for the overlap between the electromagnetic field and the MQW stack, *f*_*w*_ is the so-called electronic overlap which expresses the portion of the stack which is optically active, i.e. the ratio between the extension of the well regions and that of the whole structure, and ${\tilde{\nu }}_{p}$ is the ISB plasma frequency that we have already introduced. With the parameters retrieved from the fitting of the data points to the secular equation, it is possible to make an estimate of the Rabi frequency ${\tilde{\nu }}_{R}=\sqrt{\Gamma f_{w}}{\tilde{\nu }}_{p}/2$ and of the corresponding splitting which turns out to be around 185 cm^−1^ (23 meV). This value is comparable to or even higher than those which have been reported in the literature for III–V semiconductor alloys in the MIR [4], [10], [42].

As expected, because of the presence of the microcavity, the simulations predict the appearance of the splitting which is the typical fingerprint of the strong coupling regime [43]. Moreover, when the splitting is comparable with the matter excitation energy, i.e. ${\tilde{\nu }}_{\text{ISB}}$ , the system enters the ultra-strong coupling regime and a whole new set of quantum phenomena becomes relevant [44]. In our case, however, if we compare the Rabi frequency with the resonance frequency of the ISB transition, we only obtain ${\tilde{\nu }}_{R}/{\tilde{\nu }}_{\text{ISB}}≃8\%$ and therefore we do not expect any significant deviation from what can be predicted within our classical simulation approach.

## 4 Conclusions and perspectives

In this work we explored by means of FDTD numerical simulations the interaction between ISB transitions and metal-dielectric microcavities in a group-IV material platform, with the goal of exploring the perspective opened by the use of a heavily-doped SiGe mirror. In the end, we came to the conclusion that the investigated system, despite the relatively large mirror losses, is able to enter the strong coupling regime and a clear splitting between the two polaritonic peaks was indeed obtained; however, the splitting is not large enough to make the system enter the ultra-strong coupling regime. Further improvements in the coupling can be envisaged by acting either on the MQWs design, e.g. properly exploiting collective effects and/or Fabry–Pérot resonances in the MQW slab, or on the properties of the n-doped SiGe mirror. In this latter case, in particular, we would like to have a reflectivity which is as flat and high as possible in the region of interest in order to reduce the cavity losses. This poses a challenge when we are targeting an ISB transition centred at 1170 cm^−1^ because of the doping level which would be required to move the plasma edge at even higher energy; nevertheless, by designing a new heterostructure characterized by a lower ISB transition energy, the aforementioned goal should be achieved with reasonable doping levels. In addition, we could modify the design of the heterostructure in such a way that a Ge-like Si_1-*x*_Ge_*x*_ mirror layer, i.e. with *x* > 0.85, can be used as virtual substrate so that the effective mass of the electrons would decrease from 0.26 *m*_*e*_ to 0.12 *m*_*e*_ and the plasma frequency would increase for the same electron density. As a consequence, the electron density required to get the desired mirror-like behaviour would decrease and would be simpler to be actually achieved.
